# The Proteome of the Murine Presynaptic Active Zone

**DOI:** 10.3390/proteomes2020243

**Published:** 2014-04-24

**Authors:** Melanie Laßek, Jens Weingarten, Walter Volknandt

**Affiliations:** Institute for Cell Biology and Neuroscience, Department Molecular and Cellular Neurobiology, Max von Laue Str. 13, 60438 Frankfurt am Main, Germany; E-Mails: Lassek@bio.uni-frankfurt.de (M.L.); Weingarten@bio.uni-frankfurt.de (J.W.)

**Keywords:** presynaptic active zone, proteome

## Abstract

The proteome of the presynaptic active zone controls neurotransmitter release and the short- and long-term structural and functional dynamics of the nerve terminal. The proteinaceous inventory of the presynaptic active zone has recently been reported. This review will evaluate the subcellular fractionation protocols and the proteomic approaches employed. A breakthrough for the identification of the proteome of the presynaptic active zone was the successful employment of antibodies directed against a cytosolic epitope of membrane integral synaptic vesicle proteins for the immunopurification of synaptic vesicles docked to the presynaptic plasma membrane. Combining immunopurification and subsequent analytical mass spectrometry, hundreds of proteins, including synaptic vesicle proteins, components of the presynaptic fusion and retrieval machinery, proteins involved in intracellular and extracellular signaling and a large variety of adhesion molecules, were identified. Numerous proteins regulating the rearrangement of the cytoskeleton are indicative of the functional and structural dynamics of the presynapse. This review will critically discuss both the experimental approaches and prominent protein candidates identified. Many proteins have not previously been assigned to the presynaptic release sites and may be directly involved in the short- and long-term structural modulation of the presynaptic compartment. The identification of proteinaceous constituents of the presynaptic active zone provides the basis for further analyzing the interaction of presynaptic proteins with their targets and opens novel insights into the functional role of these proteins in neuronal communication.

## 1. Introduction

**Figure 1:**
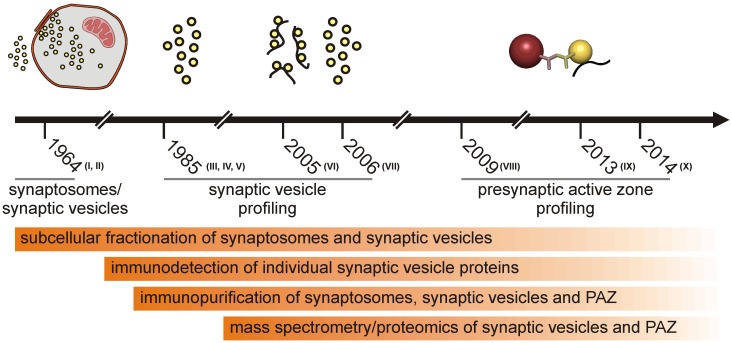
Schematic illustration highlighting historical steps leading to the isolation of the presynaptic active zone (PAZ).

Half a century of subcellular fractionation of brain tissue and protein identification culminated in the identification of the proteome of synaptic vesicles and the presynaptic active zone from murine brain. Several articles that described the isolation and global analysis of the presynaptic compartment have been published recently. Therefore, we think it is timely to review the methodology progress leading to the presynaptic active zone (PAZ) proteome discovery from rat [1,2,3] and mouse brain [4]. The purification of the presynaptic active zone was preceded by subcellular fractionation of metabolically intact nerve endings, named synaptosomes [5], which had already been reported in the early sixties of the last century [6,7]. It is beyond the scope of this review to introduce all individual experimental steps eventually leading to highly purified fractions of the presynaptic compartment comprising the active zone. The general strategy for the purification of synaptic vesicles and vesicles attached to the presynaptic plasma membrane (PAZ) is illustrated in historical steps (Figure 1). In this overview, we will focus on the composition and mode of action of the presynaptic active zone. For specific aspects of the release sites, the reader is referred to recent reviews [8,9,10,11,12,13]. The progress in the profiling of synaptosome proteomics have been reviewed in detail by Bai and Witzmann [14]. Proteomic analysis of synaptosomes derived from mouse brain identified between 1,131 [15] and 2,980 unique proteins, including 118 phosphoproteins [16]. Synaptic vesicles that play a crucial role in the purification of the active zone can be isolated from hypoosmotically disrupted synaptosomes [6,17]. They represent key organelles of chemical signaling, allowing neurons to communicate with each other and neighboring cells. Vesicle integral or membrane-associated proteins mediate the various tasks the organelle fulfills during its lifecycle. These include organelle transport, interaction with the nerve terminal cytoskeleton, uptake and storage of low molecular weight constituents and regulated interaction with the presynaptic plasma membrane at the active zone. Advances in membrane protein separation and mass spectrometry allowed the detailed description of the synaptic vesicle proteome, making synaptic vesicles the best characterized organelles (reviewed in [18]). During exo- and endo-cytosis, synaptic vesicles are tightly bound via a quadruple helical SNARE complex to the presynaptic plasma membrane [19]. This allows the immunopurification of the active zone employing antibodies directed against a cytosolic epitope of membrane integral vesicle proteins. Advanced mass spectrometry identified the proteome of these release sites [1,2,3,4]. Identified proteins include synaptic vesicle proteins, components of the presynaptic fusion and retrieval machinery, proteins involved in intracellular and extracellular signaling, a large variety of adhesion molecules and proteins potentially involved in regulating the functional and structural dynamics of the presynapse. Here, we discuss recent information concerning the proteome of the presynaptic active zone derived from mouse brain focusing on those proteins that are potentially involved in the short- and long-term structural regulation of the mature presynaptic compartment.

### 1.1. Subcellular Fractionation of the Presynaptic Active Zone

For proteomic analyses, it is of uttermost importance to reduce sample complexity as much as possible, and care should be taken to avoid contaminating compartments. Routinely, the isolation of the presynaptic active zone from murine brain starts with the enrichment of synaptosomes using the colloidal silica particles, Percoll [20], or the hydrophilic polysaccharide, Ficoll [21], both possessing low viscosity and osmolarity for density gradient centrifugation. Boyken and coworkers [3] subjected synaptosomes to limited proteolysis, employing trypsin to reduce postsynaptic contaminations. The crude synaptosome fraction is then subjected to hyposmotic lysis and sucrose density gradient centrifugation [5,6]. Following centrifugation, synaptic vesicles display a bimodal distribution, leading to the separation of synaptic vesicles in the lighter fractions and synaptic vesicles docked to the presynaptic plasma membrane in the denser fraction. This provides the opportunity to further separate these two compartments [1,2,3,4]. For further purification of these synaptic vesicle-containing fractions, immunopurification employing antibodies directed against a cytosolic epitope of membrane integral synaptic proteins has become the method of choice. Prominent targets for immunoisolation were the following synaptic vesicle proteins: the 12 membrane span, SV2 [1,2,18,22,23], the tetraspan synaptophysin [3,24] and the vesicular GABA and glutamate transporter, vGluT1 [3,25]. The antibodies were conjugated to either magnetic beads, protein A-Sepharose or methacrylate microbeads as solid support. Using the SV2 antibody for immunoisolation, the purity of the synaptic vesicles and synaptic vesicles docked to the presynaptic plasma membrane was unambiguously demonstrated by electron microscopy, marker protein analysis [1,2,26] and correlation profiling [4,23]. 

I, de Robertis *et al*., 1963 [27]; II, Whittaker *et al*., 1964 [5]; III, Ueda and Greengard, 1977 [28]; IV, Buckley and Kelly, 1985 [29]; V, Jahn *et al*., 1985 [30]; VI, Morciano *et al*., 2005 [1]; VII, Takamori *et al*., 2006 [31]; VIII, Morciano *et al*., 2009 [2]; IX, Boyken *et al*., 2013 [3]; X, Weingarten *et al*., 2014 [4].

In several studies, the immunoisolated proteins were further separated by SDS-PAGE [2,23], by two-dimensional benzyldimethyl-*n*-hexadecylammonium chloride 16-BAC-SDS-PAGE [2,23] or double SDS polyacrylamide gel electrophoresis [23] prior to protein identification by the mass spectrometry of individual protein spots. Two-dimensional BAC-SDS-PAGE was employed in combination with difference gel electrophoresis (DIGE) to analyze changes in protein abundance and posttranslational modifications of synaptic vesicle proteins under the conditions of rest and activation [32].

However, the resolution of proteins of higher molecular masses is limited in 2D gel electrophoresis, hampering their identification. In addition, the acidic buffer in BAC-SDS-PAGE interferes with the identification by mass spectrometry [23]. If prefractionation methods are employed in order to differentiate between soluble and membrane-bound proteins, care should be taken, as these substances might interfere with the subsequent identification by mass spectrometry. To overcome these limitations, immunopurified proteins often were directly solubilized in the respective buffer and subjected to ESI-MALDI-TOF/TOF.

### 1.2. Proteomic Approaches

The methodological background applied in proteomic investigations, in particular in the neuroproteomics of the synapse, has been recently discussed elsewhere [18,33,34]. The principle route and guidelines to be considered are the following: the modus operandi of choice in proteome research is to keep the number of proteins to be identified as small as possible. The appropriate proteomic strategy therefore is to enrich particular subcellular structures by subcellular fractionation. Whenever possible, efforts should be taken to purify the respective target proteome by immunoisolation. One advantage of subcellular proteomic approaches is that the identified proteins can be directly assigned to defined compartments. Especially for novel proteins, this can provide valuable information for the further evaluation of their functional role in concert with other components of an interactome [33]. 

Hydrophobic membrane proteins are technically challenging for proteomic approaches. Their amino acid sequences contain only a few cleavage sites for a vast variety of proteases, impairing their identification using peptide mass fingerprinting [35,36]. The identification procedure, especially for small hydrophobic peptides, has been substantially improved recently by employing a methanolic porcine pancreatic elastase for protein digestion or chymotrypsin instead of trypsin to increase transmembrane coverage [4,37,38]. 

### 1.3. The Active Zone Is a Dynamic Focal Hot Spot

Using the experimental protocols outlined above, the proteome of the presynaptic active zone derived from rat [1,2,3] and mouse brain [4] have been characterized in great detail. The proteome of synaptic vesicles has recently been reviewed [18] and will not be discussed here. 

**Figure 2:**
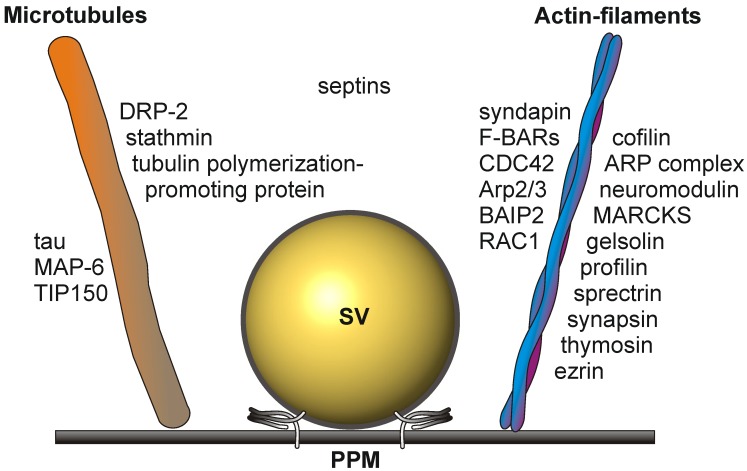
Schematic drawing depicting the cytoskeletal elements involved in the dynamic structural rearrangements of the PAZ architecture. SV, synaptic vesicle docked to the presynaptic plasma membrane (PPM).

Cytoskeletal elements involved in transport and structural dynamics are abundant at the presynaptic active zone (Figure 2). The cytomatrix of the active zone (CAZ) protein bassoon is an established constituent of the active zone [39]. Interactions between actin filaments and dynamic microtubules are required for presynaptic plasticity and dynamics [40,41]. They play a crucial role in the elaboration of terminal arbors and the recruitment of presynaptic vesicles and active zone components [42]. The tubulin polymerization-promoting protein and stathmin, involved in the destabilization and disassembly of microtubules, the microtubule-associated protein, tau, and MAP-6, involved in microtubule stabilization in neurons, have been identified as constituents of the active zone proteome in mouse brain [4]. Growing microtubule plus ends have emerged as dynamic regulatory machineries in which specialized proteins, called plus-end tracking proteins, such as the microtubule plus-end tracking protein, TIP150, bind to and control the plus-end dynamics [43].

Actin filament dynamics is sustained by the concerted action of the actin-binding proteins, cofilin, the ARP complex, neuromodulin, MARCKS, gelsolin, profilin, spectrin, synapsin and thymosin. The actin-binding protein, ezrin, is a multidomain protein required for the formation of membrane ruffles on the apical pole and links actin filaments to plasma membrane proteins [44]. Ezrin might also be involved in the functional cooperation between the microtubule and actin cytoskeleton [45].

Additional players in cytoskeleton dynamics at the active zone are the brain-specific angiogenesis inhibitor 1-associated protein 2 (BAIP2), an adaptor protein that links the small G-protein CDC42 and RAC1 to cytoplasmic effector proteins mediating the reorganization of the actin cytoskeleton and membrane ruffling, respectively. BAIP2 represent an important link between membrane and cytoskeleton in the process of neuronal growth [46]. 

Septins polymerize into heterooligomeric filaments that can associate both with actin filaments and microtubules. Presynaptic septins exhibit an increase in expression from the embryonic stage to adulthood and are present in the presynaptic terminals of mature synapses. They are enriched in preparations of synaptic vesicles (reviewed in [18]) and are co-immunoprecipitated from brain tissue, implicating a functional role within the presynapse [47].

The presence of so many proteins involved in the dynamic rearrangement of cytoskeletal tracks characterizes the active zone as a dynamic focal hot spot. This is further sustained by legions of extra- and intra-cellular signaling events at the presynaptic active zone. 

The presynaptic active zone employs a vast variety of trimeric G-proteins that mediate the intracellular signaling of its receptors. The dihydropyrimidinase-related proteins (DRPs) are necessary for signaling by Class 3 semaphorins and subsequent remodeling of the cytoskeleton. They form homo- and hetero-tetramers that promote axon guidance and branching [48]. DRP-2 is expressed in adult brain, indicating the importance of the repair and regeneration of adult neurons in preexisting neuronal networks [49]. DRP-2 is mechanically linked to tubulin heterodimers to support dynamic microtubule assembly [50]. 

Chemical signaling relies on effective and accurate compensatory endocytosis. F-BAR proteins are essential for Arp2/3 complex-mediated actin polymerization [51]. Syndapin 1 binds to membranes and mediates membrane curvature via its F-BAR domain during synaptic vesicle endocytosis and is essential for normal excitatory and inhibitory synaptic transmission [52]. Syndapin 1 is putatively important for plastic changes of neuronal morphology [51].

### 1.4. Signaling Events at the Presynaptic Active Zone

The membrane-spanning Na^+^/K^+^-ATPase (NKA) antiporter is an abundant component of the presynaptic active zone. The antiporter in concert with neighboring membrane proteins might functionally affect synaptic efficacy and neural differentiation [53]. A concerted interaction of NKA with the plasma membrane Na^+^/Ca^2+^-exchanger prevents excitotoxicity by intracellular calcium overload [54]. In addition to pumping ions, NKA has a fundamental function in signal transduction (reviewed in [55,56]). The signaling pathways of NKA are independent of changes in intracellular Na^+^ and K^+^ concentrations. NKA-mediated signaling includes the activation of Src kinase, transactivation of the EGF receptor by Src, activation of Ras and p42/44 MAP kinases and the generation of reactive oxygen species by mitochondria [56]. NKA is linked to the ERK1/2 signaling pathway by protein kinase C [57], triggering CREB activation [58]. The coordinated expression of NKA and spectrin might depend on ankyrin-2 [59], which interacts with the cell adhesion molecule, neurofascin [60]. The integral cell surface-located glycoprotein, neurofascin, has been discussed in the context of cell adhesion, axon guidance and synaptogenesis [60]. Neurofascin is one of a vast variety of cell adhesion proteins at the active zone, including Thy-1, the most abundant glycoprotein of the cell surface of mature neurons [61]. Thy-1, a constituent of secretory vesicles, has a regulatory function in neurotransmitter release [62] and is involved in stabilizing the synapse [63]. The neuronal cell adhesion molecule, NCAM-1, exists both as a membrane integral and a glycosylphosphatidylinositol (GPI) anchored isoform [64]. It interacts with both the lipid-anchored BASP1 (NAP-22), which regulates the organization and morphology of the plasma membrane [65], and neuromodulin (GAP-43) [65], which plays a general role in nerve sprouting and axon elongation [66]. Additional cell adhesion molecules at the presynaptic active zone include contactin-1, which controls neurite outgrowth and fasciculation [67], and neuroplastin, which is potentially involved in long-term potentiation at hippocampal excitatory neurons and in synaptic plasticity [68]. Additional cell adhesion molecules at the presynaptic active zone are the immunoglobulin superfamily member 8 (IgSF8), which participates in maintaining the neural network in the adult brain [69], the calcium-dependent cell adhesion protein, addherin-13 [70], the integrin-associated protein, IAP/CD47 [71], and the GPI-anchored neural cell adhesion molecule, neurotrimin [72]. The cell adhesion molecules, SynCAM 2/3, engaged in homo- and hetero-philic interactions with other nectin-like family members, are important for synapse organization, providing regulated trans-synaptic adhesion [73].

### 1.5. The Role of Calcium at the Presynaptic Active Zone

**Figure 3:**
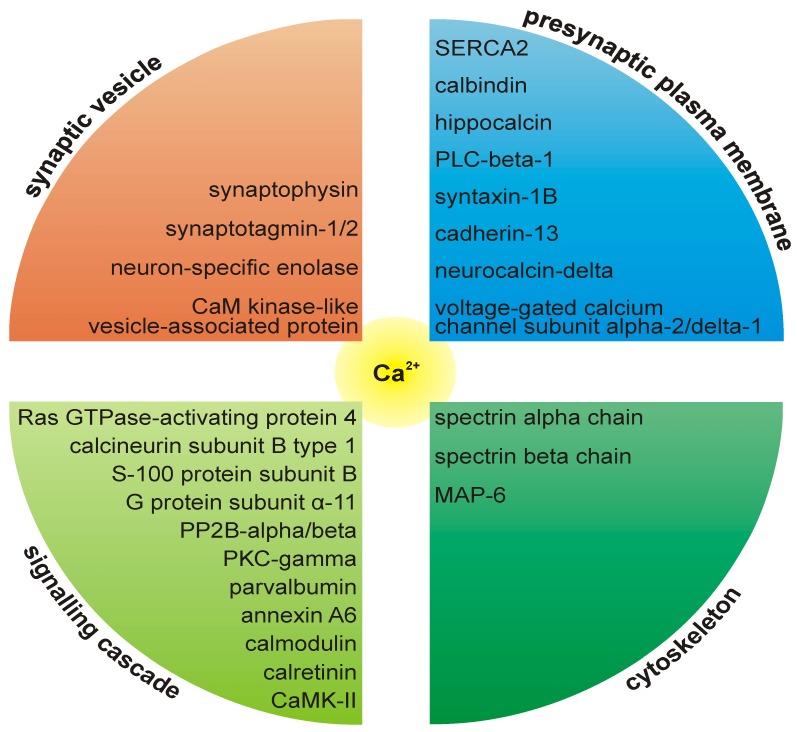
A large variety of calcium-binding proteins are present at the release sites.

The plasma membrane calcium-transporting ATPase (PMCA) has been assigned to the proteome of the presynaptic active zone [2,3]. This is in agreement with a report demonstrating that at presynaptic nerve terminals, PMCA is clustered at the active zone [74]. A large variety of calcium-binding proteins are present at the release sites, indicating the importance of calcium homeostasis at this subcompartment of the nerve terminal (Figure 3.). Calcium-activated calmodulin regulates a large variety of enzymes, ion channels and other proteins, including CaM kinases and phosphatases. The affinity of calmodulin for calcium is regulated by neurogranin, which is involved in synaptic plasticity and spatial learning [75]. Annexins are members of a group of calcium/lipid binding proteins that can dock to synaptic plasma membranes in a reversible manner [76] and can also bind to filamentous actin, membrane ruffles and focal contacts [77]. Spectrin interacts with calmodulin in a calcium-dependent manner and is a central candidate for the calcium-dependent movement of the cytoskeleton at the membrane [78].

The identification of mitochondrial proteins in all studies presumably reflects a true physical association of mitochondria with the presynaptic active zone via interaction with cytoskeletal elements. In response to neuronal stimulation, synaptic mitochondria redistribute and enhance their activity [79], suggesting that sustained neurotransmission is an energetically demanding process (reviewed in [80]). In addition to the aerobic production of ATP, mitochondria regulate Ca^2+^ concentrations [81] and have been implicated in certain forms of short-term synaptic plasticity by buffering Ca^2+^ at synapses [82,83,84,85]. Mitochondria were found to be bound to the active zone in a mitochondria-associated adherens complex [86]. Electron microscopic analysis of the immunopurified PAZ supports the notion that the presynaptic proteome includes mitochondria occasionally attached to the active zone [2]. Moreover, electron tomography revealed an elaborate cytoskeletal superstructure that connected a subset of mitochondria to the presynaptic membrane near active zones [87]. An example for a PAZ-associated mitochondrial protein with a strict neuronal expression is NIPSNAP1 [88]. It is expressed exclusively in neurons, including pyramidal neurons in the cerebral cortex, Purkinje neurons in the cerebellum, motor neurons in the spinal cord, dopaminergic neurons in midbrain and noradrenergic neurons in the brainstem.

Numerous mitochondrial proteins have been allocated to the presynaptic plasma membrane [89,90,91,92,93]. The mitochondria-located presumptive chaperone proteins, prohibitin-1 and -2, have been identified as constituents of the presynaptic active zone in rat [2] and mouse [4]. Similarly, the three isoforms of the voltage-dependent anion-selective channel protein (VADAC1–3) have been detected in both proteomes. VDACs or porins represent integral membrane proteins of about 35 kDa forming a pore in the outer mitochondrial membrane and are permeable to solutes of molecular mass <2 kDa, such as ATP, ADP and Ca^2+^ [94]. In addition to mitochondria, glycolytic enzymes that are transiently associated with synaptic vesicles provide the energy for the proton-pumping ATPase to allow the fast reuptake of neurotransmitter after exocytosis. Glycolytic enzymes have been consistently assigned to the synaptic vesicle proteome (reviewed in [18]). 

## 2. Additional Protein Constituents of PAZ

Mass spectrometry identified bassoon [3,4] and piccolo, rab-interacting molecule (RIM), liprin-α, CASK and ERC (ELKS/Rab6-interacting/CAST) [3] as constituents of the cytomatrix of the active zone (CAZ; [95]). Some of these proteins presumably exist in low numbers at the release sites and can easily escape identification by mass spectrometry. Therefore, we expect additional key players to be identified in the future. Interestingly, the amyloid precursor protein family members (APP; APLP1 and APLP2) were identified as constituents of the presynaptic plasma membrane at release sites in murine brain [26].

## 3. Conclusions

Refinement of subcellular fractionation, immunopurification and mass spectrometry culminated in the isolation and identification of the proteinaceous inventory of the subcellular compartment containing synaptic vesicles docked to the presynaptic plasma membrane, comprising the presynaptic active zone. These release sites have been purified from murine brain by subcellular fractionation and subsequent immunopurification employing antibodies directed against a cytosolic epitope of membrane integral synaptic vesicle proteins. The purity of the active zone has been confirmed by electron microscopy, marker protein analysis and correlation profiling.

Mass spectrometric analyses characterize the proteome of the presynaptic active zone derived from murine brain, implicating considerable functional and structural dynamics of the release sites. The active zone can indeed be considered as a highly active subcompartment of the presynapse. The identification of the participating protein components is a prerequisite for further functional investigations and provides the basis for evaluating their interaction. Novel proteins not previously assigned to this compartment can be placed in this mosaic to create a functional scenario of neuronal communication and plasticity in these dynamic focal hot spots. Taken together, these data further support the notion that not only the developing, but also the adult, nerve terminal is a highly dynamic compartment capable of undergoing continued structural and functional changes.

## Abbreviations

PAZ: presynaptic active zone
PPM: presynaptic plasma membrane
SV: synaptic vesicle
